# Percutaneous Sonazoid-enhanced ultrasonography combined with in vitro verification for detection and characterization of sentinel lymph nodes in early breast cancer

**DOI:** 10.1007/s00330-020-07639-2

**Published:** 2021-01-27

**Authors:** Yunxia Hao, Yan Sun, Yutao Lei, Hongmei Zhao, Ligang Cui

**Affiliations:** 1 Department of Ultrasound, Peking University Third Hospital, 49 North Garden Road, Haidian District, Beijing, 100191 China; 2 Department of General Surgery, Peking University Third Hospital, 49 North Garden Road, Haidian District, Beijing, 100191 China

**Keywords:** Sentinel lymph node, Sonazoid, Contrast media, Ultrasonography, Breast neoplasms

## Abstract

**Objectives:**

To assess the efficacy of percutaneous Sonazoid-enhanced ultrasound and in vitro verification for identification sentinel lymph nodes (SLNs) and diagnosis of metastatic SLNs in patients with early breast cancer (BC).

**Methods:**

Sixty-eight patients with early BC were enrolled finally. After the induction of general anesthesia, 0.4 ml of Sonazoid (SNZ), a new second-generation tissue-specific ultrasound contrast agent (UCA), mixed with 0.6 ml of methylene blue, was injected intradermally. The lymphatic vessels and connected SLNs were immediately observed and marked. After being resected, these SLNs were soaked in saline water and examined still in the mode of contrast-enhanced ultrasound (CEUS) in vitro. This procedure could ensure that all the enhanced nodes had been removed as much as possible. The numbers of SLNs detected by UCA and blue dye were recorded. The enhancement patterns of SLNs were compared with the final pathological results.

**Results:**

SLNs detection rate by SNZ-CEUS was 100%, which was higher than that by blue dye (95.59%). CEUS identified a median of 1.5 nodes, while blue dye identified a median of 1.9 nodes per case (*p* = 0.0012). When homogeneous high perfusion and complete annular high perfusion were regarded as negative nodes, the sensitivity and negative predictive value were 92.31% and 96.79% respectively, while the specificity was 84.21%.

**Conclusions:**

Percutaneous SNZ-enhanced ultrasonography combined with in vitro verification is a feasible and reliable method for SLNs identification intraoperatively. Enhancement patterns can be helpful in determining the status of SLNs.

**Key Points:**

*• CEUS with percutaneous injection of Sonazoid can successfully identify SLNs with the rate of 100% in early breast cancer patients, higher than 95.59% of blue dye.*

*• Sonazoid, with high affinity with reticuloendothelial cells, increases the imaging time of SLNs and facilitates biopsy intraoperatively better than Sonovue as a lymphatic tracer.*

*• Homogenous high and complete annular high perfusions have a sensitivity of 92.31% and a negative predictive value of 96.79% in the prediction of uninvolved SLNs.*

## Introduction

Breast cancer (BC) has the highest incidence among cancers in women, both in China and in other countries worldwide [1]. Other than the tumor’s biology and anatomical staging, axillary staging is of great importance for the treatment and prognosis of BC. In the past, axillary staging was based on the histopathological results of axillary lymph node dissection (ALND) [2], which was associated with a significant risk of complications, such as lymphedema, arm paresthesia, and shoulder motion restriction. Axillary staging surgery has evolved from ALND to less-morbid sentinel lymph node biopsy (SLNB) in early BC patients since 2005. SLNB can accurately predict axillary lymph nodes’ status and ensure that only patients with positive SLNs will receive ALND [3].

Many methods have been developed for sentinel lymph node (SLN) detection in the procedure of SLNB. The first-line procedure recommended is the joint use of blue dye and radioisotope. However, the radioisotope is expensive and involves radiation, which limits its widespread use, especially in developing countries and basic hospitals. Blue dye is inexpensive and it alone nowadays has been the most widely used alternative method in China and globally [4]. Notably, however, the identification rate by blue dye alone is variable, which ranges from 66 to 94%, and the false-negative rate ranges from 0 to 12% [5]. Moreover, skin necrosis and anaphylactic relations to the dye have been reported [6]. Therefore, the constraints of the existing biopsy methods indicate the urgent need for the development of novel alternative methods for localizing SLNs.

In addition, SLNB are proved to be pathologically negative in nearly up to 74% of patients [7]. Still even SLNB may carry side effects such as lymphedema (2–6%) [8] and arm paresthesia (9%) [9], and it is time consuming and complex to implement, especially for less-trained surgeons. Therefore, an approach that is less invasive and easier than SLNB and that offers equal survival outcome with SLNB is warranted to assist in characterizing the status of axillary lymph nodes (ALNs).

Intradermal contrast-enhanced ultrasonography (CEUS) is a novel, noninvasive, portable, and real-time technique that has recently been introduced for identifying and characterizing SLNs. This technique was initially used in a swine melanoma model in 2004 [10], and the human study in BC was first reported by Omoto et al in 2006, who used 25% albumin as the ultrasound contrast agent (UCA) [11]. Many studies have confirmed its safety in intradermal injection in early BC patients [12, 13]. However, the performance of this procedure varied across previous studies. The SLN identification rate was between 70 and 100% [14–17] and the diagnostic accuracy, particularity the specificity, of CEUS for determining SLN status requires improvement [18]. On the one hand, improvement and standardization of the administration of percutaneous CEUS are strongly required to ensure successful identification of SLNs. On the other hand, different types of contrast agents used in previous studies may have various effects in the identification and characterization of SLNs. Sonovue (Bracco Imaging SpA) was the most commonly used UCA in previous studies, but its mechanical properties limit its use with high-frequency linear array probes for breast scanning and its capacity for long-term imaging. Recently, a more stable and tissue-specific contrast agent, named Sonazoid (SNZ) (GE Healthcare), has been introduced, mainly for the evaluation of the liver and mammary glands. It is a perflubutane microbubble that was stabilized using hydrogenated egg phosphatidylserine sodium [19, 20]. A higher resistance to ultrasound mechanical index (MI) and the ability to be engulfed by reticuloendothelial cells in lymph nodes [10, 21] make SNZ be of major interest, as it appears to hold promise as constituting a more reliable SLN tracer than Sonovue in early BC patients.

Therefore, the aims of this preliminary study were to introduce a more reliable procedure of trans-lymphatic CEUS using SNZ and validate its effectiveness for the intraoperative localization of SLNs as well as to explore its potential value in the diagnosis of SLNs status.

## Materials and methods

### Patients

Between June 2019 and February 2020, 77 consenting patients who were pathologically diagnosed with BC and were scheduled for SLN biopsy and lumpectomy or mammectomy were consecutively recruited for the study. The exclusion criteria were as follows: ALN involvement which was proved positive previously, a history of protein allergy, inflammatory BC, stage IV BC, and a history of neoadjuvant chemotherapy. All patients who participated in this research signed an informed consent form, and the study was approved by the ethics committee of Peking University Third Hospital (IRB approval number is M2017394). Finally, 68 patients were recruited for the study, 8 patients were excluded because of the history of neoadjuvant chemotherapy, and 1 patient because of lacking pathology results.

All examinations were implemented by GE LOGIQ E9 general imaging system (GE Healthcare) equipped with a high-frequency linear array probe (ML6-15) and dedicated contrast pulse sequences (CPS). The MI was set to 0.2, and the focus zone was adjusted to a depth of 10–50 mm. The SNZ contrast agent was a dry powder, which needed to be prepared by 2 ml of sterile saline into microbubbles. Methylene blue (Jumpcan Pharmaceuticals) was used as the blue dye.

### CEUS procedure

A gray-scale ultrasound examination of ALNs was given first. The sizes and thickness between the capsule and hilum of the lymph nodes were recorded. Immediately after induction of general anesthesia and before the SLNB, the microbubble suspension (0.4 ml), mixed with 0.6 ml of methylene blue, was intradermally injected into the upper outer quadrant around the areola by means of a plastic syringe with a 22G needle. In most cases, hyperechoic subcutaneous lymphatic channels directing to the axillary could be detected immediately after the injection. If not, massaging the areola for 15–30 s could help the draining. When the lymphatic drainage pathway was not displayed successfully, another injection was administered. The case was regarded as a failure when lymphatic vessels still did not fill after 2 consecutive injections. Live dual images were shown synchronously to confirm the presence of the SLNs, although sometimes it was difficult to recognize lymph node architecturally in the gray-scale pattern.

Once identified, the locations of SLNs were marked with a pen, and the size, depth (perpendicular to skin), and enhancement patterns of SLNs were recorded on site.

All sonographic examinations were performed by 1 sonologist. And another experienced sonologist gave an independent result by reviewing the loops. Both the two sonologists were blinded to the patients’ clinical data and pathological results.

**Fig. 1:**
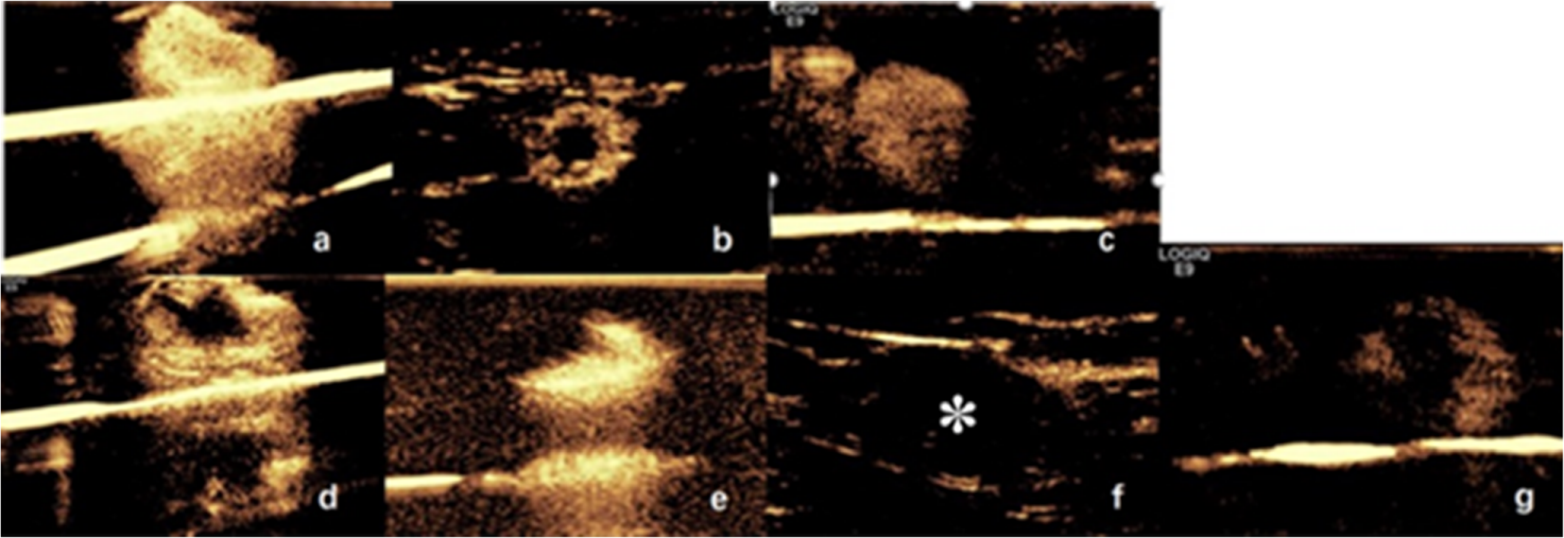
Different types of enhancing patterns, **a** Type I, bright and homogenous or brush-like perfusion; **b** Type II, complete annular high enhancement with no perfusion in the central area which is in accordance with the hilum of lymph nodes; **c** Type III, uniform but low perfusion; **d** Type IV, inhomogeneous high enhancement with a clearly defined but irregular area of no enhancement; **e** Type V, incomplete annular high enhancement; **f** Type VI, no enhancement, white asterisk indicates the SLNs with no perfusion of contrast agent; **g** Type VII, low and inhomogeneous enhancement

The patterns of enhancement of SLNs could be divided into 7 types (Fig. 1): type I: homogenous high enhancement; type II: complete annular high enhancement; type III: homogenous low enhancement; type IV: inhomogeneous high enhancement; type V: incomplete annular high enhancement; type VI: no enhancement; and type VII: inhomogeneous low enhancement.

Type III was not recorded in previous studies, and various reasons can lead to homogenous low enhancement in theory [22–24]. For example, patients’ age, physique, lymphatic vessel diameter, lymphatic flow, the benign proliferation of lymphatic follicles or the lymphatic sinus, and the increasing intranodal pressure caused by a little tumor volume in SLNs may slow the transit time of SNZ and decrease the amount of SNZ gathering in SLNs. It is hard to say whether type III is more likely to be a positive predictor or a negative predictor. So we made 2 classification methods: by one method, type I, II, and III SLNs were considered non-involved nodes, and the remaining types were considered involved nodes, while the other, type I and II SLNs were considered negative, and the rest were considered positive.

### SLNB procedure and in vitro verification

After the localization procedure, conventional surgery area disinfection and draping were implemented. A 2–3-cm incision line was determined under the guidance of the skin marker made by the sonologist. Blue-stained lymph nodes or blue-stained lymphatic vessels leading to lymph nodes were considered SLNs. During SLNB, clinically palpable lymph nodes were also excised and categorized as axillary samples.

All excised SLNs and axillary samples, soaked in normal isotonic physiologic saline, were reevaluated in vitro by CEUS. These lymph nodes were examined to see whether they were those SLNs imaged previously in vivo. Assessment indicators included the dimension, the depth, the morphology of lymph nodes (such as the shape, cortical thickening, and fatty hilum of lymph node) in gray-scale ultrasonic images, and the enhancement pattern in CEUS images.

Then nodes were submitted to standard histological analysis, and the number of each category of ALNs was recorded for each patient. All ALNs were evaluated by immuno-histochemistry (IHC).

### Statistical analysis

The identification rate was defined as the proportion of patients with SLNs identified by either UCA or methylene blue. Differences among categorical variables were analyzed with a chi-square test. A paired *t* test was used to compare means of continuous normally distributed data. Cohen’s kappa was used to assess inter-radiologist agreement over enhancing patterns of lymph nodes. The agreement was classified as follows: excellent, kappa > 0.80; good, kappa = 0.61–0.80; moderate, kappa = 0.41–0.60; fair, kappa = 0.21–0.40; and poor, < 0.20. Areas under the receiver operating characteristic (ROC) curve (AUCs) were compared for two different classification methods with the help of MedCalc (version 19.07, MedCalc Software bvba). The SPSS software (version 20, SPSS Inc.) was used for statistical analyses and *p* < 0.05 was considered to be statistically significant difference.

## Results

### Baseline patient characteristics

We included 68 female patients with a median age of 54 years (range, 26–77 years) in this study. The clinical features of these patients are presented in Table 1. The tumor sizes of 2 patients were unknown because the breast lump had been removed in other hospitals.

**Table 1:** Basic characteristics of patients

Characteristic	Patients
Tumor size	
Tis	7
T1	23
T2	27
Multi-focal	9
Tumor histology	
DCIS	6
Paget disease	1
IBC	53
Others	6

*Note*: *DCIS* ductal carcinoma in situ, *IBC* invasive breast carcinoma

There are 2 patients that lack precise information about tumor size and histology, so they were not included in Table 1

### Identification rate of SLNs by CEUS

The rate of SLN identification by CEUS with intradermal injection of SNZ in the recruited patients was 100% (68/68). The identification rate by use of blue dye only was 95.59% (65/68). Overall, 102 SLNs were detected by SNZ-CEUS, with a median number of 1.5, and 139 SLNs were detected by blue dye, with a median number of 1.9 (*p* = 0.0012). More SLNs were recognized by blue dye than by SNZ in most cases, while SNZ detected more SLNs than blue dye in 6 patients (8.82%). The total number of ALNs excised was 229, with 3.37 ALNs per case.

### Enhancement pattern of SLNs

Pathological results of the 102 SLNs detected by SNZ-CEUS are depicted in Table 2.

**Table 2:** Enhancing patterns and pathological status of SLNs

	Enhancement of SLNs							
Pathology	I	II	III	IV	V	VI	VII	Total
Positive	2	0	1	13	2	7	1	26
Negative	58	6	5	2	2	1	2	76
Sub total	60	6	6	15	4	8	3	102

We found that type I nodes were the most typical pattern in negative nodes (58/76, 76.32%), and types IV and VI were more common in positive nodes (13/26, 50%; 7/26, 26.92%). The diagnosis coincidence rate, sensitivity, specificity, positive predictive value, negative predictive value, false-negative rate, and false-positive rate of these 2 classification methods for determining SLN status are presented in Table 3. It showed no statistical difference between the diagnostic accuracy of these two classification methods (*p* = 0.6107). For the first classification method, the AUC was 0.888 (95% CI: 0.810–0.942). For the second classification method, the AUC was 0.876 (95% CI: 0.795–0.933).

**Table 3:** Diagnostic indexes of enhancing types by the 2 different classification methods

Diagnostic index	Classification 1	Classification 2
Coincidence rate	90.02% (92/102)	86.27% (88/102)
Sensitivity	88.46% (23/26)	92.31% (24/26)
Specificity	90.79% (69/76)	84.21% (64/76)
Positive predictive value	76.67% (23/30)	66.67% (24/36)
Negative predictive value	95.83% (69/72)	96.97% (64/76)
False-negative rate	11.54% (3/26)	7.69% (2/26)
False-positive rate	9.21% (7/76)	15.79% (12/76)

The two readers agreed with each other on the classification of enhancing patterns in 91 out of 102 nodes. The *k* coefficient was 0.823, indicating good inter-reader agreement. The reproducibility of the classification of enhancing patterns of CEUS with SNZ is high in this study.

### Safety

No serious side effects related to the percutaneous injection of SNZ such as skin reactions around the injection site or allergic reactions were observed after surgery.

## Discussion

Percutaneous CEUS is an emerging modality for the identification and characterization of SLNs in BC patients. To date, the contrast agent mostly being used for the identification of SLNs was Sonovue (Bracco Imaging). The failure rates of SLN detection by intradermal injection of Sonovue range between 1.8 and 11% [18, 25–29]. SNZ has gained more attention in recent years. Although it has been mostly used for hepatic lesions [30], it was only introduced for SLN in BC patients in a few studies with limited sample sizes [31–33]. A study in Japan with 20 patients conducted by Omoto et al first reported a detection rate of 70% with SNZ, which was much lower than that of the γ-probe-guided method (100%), reported in 2009 [31]. However, the rate increased to 100% in 2015, in a report based on 32 patients [32], and to 98% in 2017 in a report based on 100 patients [33], which was similar to that of blue dye and/or radiocolloid. In this study, we have demonstrated that percutaneous CEUS, used in combination with SNZ, showed good performance in mapping lymphatic drainage and detecting SLNs in early BC patients with a rate as high as 100%, which was better than that of blue dye (95.59%) in our study.

It is worth noting that several previous studies only marked the locations of SLNs in the skin when they were identified by CEUS before SLNB. This design has an obvious drawback: during the surgical procedure of SLNB, there is a very definite possibility of relative displacement between the nodes and the skin mark, which makes it difficult for surgeons to find the true CEUS-SLNs especially in women with axillary accessory breast tissue. A serious, easy, and convenient procedure for CEUS with SNZ was developed in our study to solve this problem.

First, when it is difficult for surgeons to find the SLNs intraoperatively, we can relocate the SLNs with CEUS in real time, simply with the help of a disposable aseptic laparoscopic sleeve. SNZ is stable and has a long lifespan in vivo [34] and is taken up by the reticuloendothelial cells within the lymph nodes, providing signal enhancement for up to 24 h [12, 13], facilitating imaging at any time during the operation. In contrast, Sonovue imaging can only last 4 min [26, 28]. It has been declared that the phosphatidylserine membrane of SNZ microbubbles gives rise to its high affinity with reticuloendothelial cells [21]. Goldberg et al confirmed the presentation of the SNZ microbubble within reticuloendothelial cells of SLNs by scanning electron microscopic evaluations, thereby explaining the prolonged retention of SNZ over Sonovue [10].

Second, neither surgeons nor sonologists can completely ensure that the excised blue-stained lymph nodes are those SLNs imaged previously by CEUS, and thus, it cannot be guaranteed that the nodes evaluated by CEUS corresponded to those evaluated by pathology. Xie et al [28] attempted to locate SLNs with guidewires after their identification by intradermal CEUS, but this procedure is inevitably invasive and time-consuming. Therefore, we conducted in vitro CEUS of the excised nodes to ensure that all contrast-enhanced SLNs seen in vivo had been excised. This additional easy and convenient step not only increased surgeons’ conviction of completely removing SLNs to reduce the false-negative rate of SLNB but also provided the possibility of assessing the one-to-one correspondence between the CEUS enhancement pattern and the pathology of isolated lymph nodes, allowing more accurate assessment of the diagnostic performance of CEUS.

**Fig. 2:**
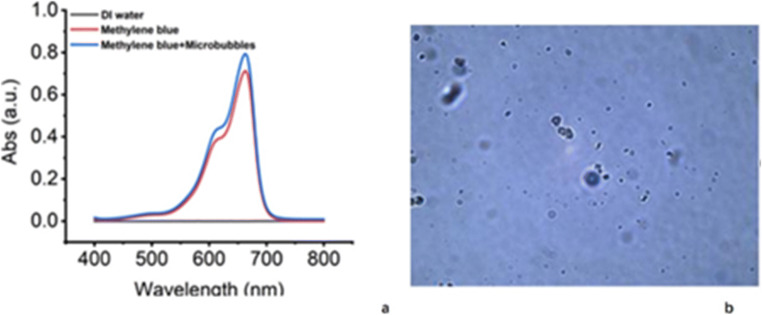
**a** There was no alternation in absorbance of methylene blue after being blended with microbubbles. **b** Confirmed integrity of microbubbles after being blended with methylene blue

For the CEUS imaging of SLNs, in addition to the reasonable choice of the type of contrast agent, the dose of contrast agent, the MI value, and frequency of the sound field should also be considered when optimizing imaging. SNZ has superior tolerance to a comparatively high acoustic pressure because of its membrane. And MI is positively related to the sound pressure, which affects the penetration of the acoustic beam. The MI was set to nearly 0.2 in the procedure of CEUS with SNZ, higher than the 0.06 or 0.07 used with Sonovue. This MI is more appropriate for deep SLNs imaging, especially for those with accessory breast, which may decrease FNR to some extent. A high-frequency probe was used, which is in accordance with routine examination of breast and axillary nodes, with a better imaging effect than achieved with a lower-frequency transducer, as used in previous studies either with SNZ or Sonovue [31, 32]. In terms of the dose of SNZ, we found that 0.4 ml is enough for imaging of SLNs in pre-experiment, lower than 2 ml used by Omoto et al [31, 32] and 1 ml recommended by Machado et al [12]. To simplify the procedure, microbubbles and blue dye were mixed and injected at the same time. No visible polymer exists after the mixture of the two solutions. The absorbance of methylene blue was not altered after being mixed up with SNZ (Fig. 2a). The integrity of the microbubbles was confirmed using a high-power microscope (Fig. 2b).

The mean number of SLNs detected by SNZ was 1.5, less than 1.9 detected by blue dye only (*p* < 0.05). The most possible cause for this finding is that blue dye as well as the radioactive colloid may pass through the SLN to other adjacent lymph nodes [35]. The actual number of SLNs varied from patient to patient. With the help of CEUS, the number can be confirmed before SLNB to guarantee an adequate removal of lymph nodes, and to avoid unnecessary resection of non-SLN.

Although SLNB for early-stage BC is the standard of care today, it is important that surgeons and radiologists move the field forward, by seeking safe ways to eliminate non-therapeutic procedures. Conventional gray-scale and color Doppler ultrasound are recommended worldwide in the current axillary pathway for patients with BC in the National Comprehensive Cancer Network (NCCN) guideline, which regrettably shows an overall low sensitivity between 26 and 76% [36]. A few prospective studies have shown that CEUS with intradermal injection of contrast agent is a helpful technique for determining SLN involvement, but the diagnostic efficiency was variable, and enhancing patterns were only divided into three simple types: homogeneous enhancement, inhomogeneous enhancement, and no enhancement [15, 18, 27, 28].

CEUS with SNZ has not previously characterized the status of SLNs in human BC patients. In this study, we found that there were 7 types of enhancement patterns. Type I was the most common pattern in negative nodes (58/76, 76.32%), and types IV and VI were more common in positive nodes (13/26, 50%; 7/26, 26.92%), which is similar to the results in Xie’s study [28]. We had two classification methods of these 7 types, but there was no statistical difference between their diagnostic accuracy (*p* = 0.6107). It is worth noting that the negative predictive value (96.79%) was higher and the FNR (7.69%) was lower by the second classification method, which confirmed that type I and II enhancements have high negative predictive value in diagnosing SLN involvement, indicating great potential to reduce the number of patients unnecessarily undergoing surgical SLNB in clinical settings in the era of axillary node conservation. However, the mechanism of the overlap between enhancing patterns of benign and malignant nodes needs to be further researched, and simply relying on the enhancing patterns cannot effectively predict node status.

There was good inter-reader agreement on the classification of enhancement patterns (k coefficient: 0.823), similar to 0.886 in a previous study by Zhao J et al [18]. Zhao J had classified enhancement patterns into three kinds, while we observed and classified enhancement patterns into 7 types, which may reduce the classification consistency. So it is still to be emphasized that a standardized definition of these seven enhancing patterns should be clarified, and an appropriate training of operation of CEUS should be done to guarantee that this technique could be readily and accurately applied to the real clinical setting.

The present study had the following limitations that deserve comment. First, the sample size should be increased and the reasons resulting to the misdiagnosis of SLN status should be figured out. Further studies including node examinations by transmission electron microscope could be implemented to clarify the relationship between enhancing patterns and pathology results and means of improving the diagnostic accuracy should be explored.

In conclusion, CEUS with intradermal injection of SNZ and in vitro verification is an easy and accurate modality to identify SLNs intraoperatively in early BC patients. The enhancing patterns on CEUS could be a promising indicator for the involvement of SLNs. Further studies should be implemented to validate the role of SNZ-CEUS and ensure that it is not inferior to SLNB as a noninvasive replacement with regard to important oncological outcomes.

## Abbreviations

ALN: Axillary lymph node
ALND: Axillary lymph nodes dissection
AUC: Areas under the ROC curve
BC: Breast cancer
CEUS: Contrast-enhanced ultrasonography
CPS: Contrast pulse sequence
IHC: Immuno-histochemistry
MI: Mechanical index
ROC: Receiver operating characteristic
SLN: Sentinel lymph node
SLNB: Sentinel lymph node biopsy
SNZ: Sonazoid
UCA: Ultrasound contrast agent
